# Measuring Creatinine Clearance Is the Most Accurate Way for Calculating the Proper Continuous Infusion Meropenem Dose for Empirical Treatment of Severe Gram-Negative Infections among Critically Ill Patients

**DOI:** 10.3390/pharmaceutics15020551

**Published:** 2023-02-07

**Authors:** Carla Troisi, Pier Giorgio Cojutti, Matteo Rinaldi, Cristiana Laici, Antonio Siniscalchi, Pierluigi Viale, Federico Pea

**Affiliations:** 1 Department of Medical and Surgical Sciences, Alma Mater Studiorum-University of Bologna, 40138 Bologna, Italy; 2 Clinical Pharmacology Unit, IRCCS Azienda Ospedaliero-Universitaria di Bologna, 40138 Bologna, Italy; 3 Infectious Diseases Unit, Department for Integrated Infectious Risk Management, IRCCS Azienda Ospedaliero-Universitaria di Bologna, 40138 Bologna, Italy; 4 Division of Anesthesiology, Department of Anesthesia and Intensive Care, IRCCS Azienda Ospedaliero-Universitaria di Bologna, 40138 Bologna, Italy

**Keywords:** continuous infusion meropenem, therapeutic drug monitoring, critically ill patients

## Abstract

Assessment of glomerular filtration rate (GFR) is necessary for dose adjustments of beta-lactam that are excreted by the kidneys, such as meropenem. The aim of this study was to compare the daily dose of 24 h-continuous infusion (CI) meropenem when GFR was calculated by means of measured creatinine clearance (mCL_CR_) or estimated by the CKDEPI (eGFR_CKDEPI_), Cockcroft–Gault (eGFR_CG_), and MDRD (eGFR_MDRD_) equations. Adult critically ill patients who underwent therapeutic drug monitoring (TDM) for the assessment of 24 h-CI meropenem steady state concentration (Css) and for whom a 24 h-urine collection was performed were retrospectively enrolled. Meropenem clearance (CL_M_) was regressed against mCL_CR_, and meropenem daily dose was calculated based on the equation infusion rate = daily dose/CL_M_. eGFR_CKDEPI_, eGFR_CG_, and eGFR_CKDEPI_ were regressed against mCL_CR_ in order to estimate CL_M_. Forty-six patients who provided 133 meropenem Css were included. eGFR_CKDEPI_ overestimated mCL_CR_ up to 90 mL/min, then mCL_CR_ was underestimated. eGFR_CG_ and eGFR_MDRD_ overestimated mCL_CR_ across the entire range of GFR. In critically ill patients, dose adjustments of 24 h-CI meropenem should be based on mCL_CR_. Equations for estimation of GFR may lead to gross under/overestimates of meropenem dosages. TDM may be highly beneficial, especially for critically ill patients with augmented renal clearance.

## 1. Introduction

Multidrug-resistant (MDR) Gram-negative pathogens are the leading cause of severe infections in critically ill patients [1]. Despite available treatments, the in-hospital mortality rate for patients with suspected or proven infections is as high as 30% [1]. Among the most important causes of antimicrobial treatment failure and worse clinical outcome in critically ill patients are the high level of antimicrobial resistance, the high inter-individual pharmacokinetic variability, and the frequent immunocompromised state [2,3].

Current Italian and European guidelines recommend the novel beta-lactams/beta-lactamase inhibitors as first-line agents for the treatment of severe infections caused by carbapenemase-producing Gram-negative pathogens [4,5]. However, meropenem still remains a valuable option in the context of extended-spectrum beta-lactamases (ESBLs)-producing Enterobacterales [6,7], as well as for susceptible strains of *Pseudomonas aeruginosa* or *Acinetobacter baumannii* [6,8].

Meropenem has time-dependent bactericidal activity, and its efficacy is related to the duration of time the serum concentration is above the minimum inhibitory concentration (MIC) of the micro-organism (time above MIC) for at least 40% of the dosing interval [9]. However, in critically ill patients and/or immunocompromised subjects, more aggressive pharmacodynamic targets of efficacy up to 100% t > 4–6 × MIC are currently advocated for maximizing efficacy [10] and preventing the development of resistance [11].

The attainment of such higher pharmacodynamic targets may be facilitated by the use of 24 h-continuous infusion (CI) administration. Considering that meropenem is mainly excreted as an unmodified drug by the renal route, the calculation of the daily dose that is necessary for attaining the pharmacodynamic efficacy target should be based on patient’s glomerular filtration rate (GFR) [12,13,14]. Measured creatinine clearance (mCL_CR_) should be approached as the best surrogate of GFR, but this could be time- and resource-consuming. That is why GFR is frequently estimated nowadays by means of validated mathematical formulas, such as the Cockcroft–Gault (CG) formula, the chronic kidney disease epidemiology collaboration (CKD-EPI) formula, and the modification of diet in renal disease (MDRD) formula. However, such formulas were not assessed and validated specifically in the critical care setting, so that estimated glomerular filtration rate (eGFR) based on them could deviate consistently from mCL_CR_, thus, leading to drug underdosing or overdosing.

The aim of this study was to evaluate whether eGFR based on CG, CKDEPI, and MDRD equations could be as reliable as mCL_CR_ or not in calculating the daily dose of 24 h-CI meropenem for properly treating nosocomial infections in a cohort of critically ill patients.

## 2. Materials and Methods

This retrospective monocentric study was conducted among critically ill patients admitted to the post-transplant Intensive Care Unit of the IRCCS Azienda Ospedaliero-Universitaria di Bologna, Italy, in the period December 2020–January 2022. All of the included patients received 24 h-CI of meropenem and underwent real-time therapeutic drug monitoring (TDM) for optimizing empirical or targeted treatment of Gram-negative infections.

The following demographic and clinical data were collected from each patient’s medical record: age, gender, weight, height, type and site of infection. Patients undergoing renal replacement therapy were excluded.

Meropenem therapy was started with a loading dose of 2 g over 2 h and continued with a maintenance dose initially based on the patient’s renal function (ranging from 1 g q6h over 6 h to 0.25 g q6h over 6 h) and subsequently optimized by means of TDM coupled with expert clinical pharmacological advice (ECPA). Stability of 24 h-CI meropenem was granted by reconstitution of the aqueous solution every 6–8 h with infusion over 6–8 h [15].

TDM of meropenem was performed within 48–72 h from the starting treatment and then reassessed every 48–72 h. Peripheral venous blood samples were centrifugated, and plasma was then separated. Meropenem plasma concentrations were analyzed by means of a liquid chromatography-tandem mass spectrometry (LC–MS/MS) commercially available method (Chromsystems Instruments & Chemicals GmbH, Munich, Germany), with a lower limit of detection of 0.3 mg/L. The desired pharmacodynamic target of meropenem efficacy was set at a steady state concentration (Css) to MIC (Css/MIC) ratio of 4–8 [13].

At each TDM assessment, mCL_CR_ (mL/min) was performed and calculated as follows:

$${\mathrm{mCL}}_{\mathrm{CR}}=\frac{U_{\mathrm{CR}}\times U_{\mathrm{Volume}}}{S_{\mathrm{CR}}\times T}$$

where U_CR_ is the urinary creatinine concentration (mg/dL), U_Volume_ is the urinary volume (mL), S_CR_ is the serum creatinine concentration (mg/dL), and T is the 24 h collection time (equal to 1440 min). Creatinine was measured both in serum and urine by enzymatic assay.

Patients with mCL_CR_ < 30 mL/min/1.73 m^2^ were defined as having an episode of acute kidney injury (AKI), whereas those with mCL_CR_ ≥ 130 mL/min/1.73 m^2^ were defined as having an episode of augmented renal clearance (ARC).

Instead, eGFR was assessed by means of three different formulas: the Cockcroft and Gault formula (eGFR_CG_) [16], the CKD-EPI formula (eGFR_CKDEPI_) [17], and the MDRD formula (eGFR_MDRD_) [18].

A multistep approach was used to assess whether the eGFR calculated by means of the aforementioned formulas could be considered as reliable as the mCL_CR_ for properly calculating the daily meropenem dosages needed for optimal treatment for the critically ill patients.

First, meropenem total clearance (CL_M_) was calculated in each single patient by means of the following equation:

$${\mathrm{CL}}_{M}=\frac{\mathrm{IR}}{C_{\mathrm{ss}}}$$

where CL_M_ is the meropenem clearance (L/h), IR is the hourly meropenem infusion rate (mg/h), and Css is the meropenem steady-state plasma concentration (mg/L).

Second, linear regression between CL_M_ and mCL_CR_ was performed.

Third, the meropenem daily dosing regimen was estimated by means of the mCL_CR_. For doing so, being meropenem IR (mg/h) = CL_M_ × Css, CL_M_ was expressed as a function of mCL_CR_ by means of following equation of linear regression: CL_M_ = a + b × mCL_CR_ (where a and b are the intercept and slope, respectively). In this way,

meropenem daily IR-mCL_CR_ (mg/24 h) = [a + b × mCL_CR_] × Css × 24

where daily IR-mCL_CR_ is the daily meropenem infusion rate (mg/24 h).

Subsequently, linear regressions between mCL_CR_ and each of the eGFR, namely eGFR_CKDEPI_, eGFR_CG_, and eGFR_MDRD_, were assessed. The resulting linear regression equations were used for estimating the meropenem daily dosing regimens based on each of the eGFR formulas (one each for eGFR_CKDEPI_, eGFR_CG_, and eGFR_MDRD_).

Accordingly:

IR-eGFR_CKDEPI_ (mg/24 h) = [c + d × mCL_CR_] × Css × 24,

IR-eGFR_CG_ (mg/24 h) = [e + f × mCL_CR_] × Css × 24, and

IR-eGFR_MDRD_ (mg/24 h) = [g + h × mCL_CR_] × Css × 24

The squared coefficient of regression (R^2^) was used to evaluate the performance of each regression. A one-way analysis of variance was used to assess differences between measured and estimated renal function and between the meropenem daily dose based on mCL_CR_ versus eGFR. All statistical analysis and plotting were performed using R (version 4.0.3).

## 3. Results

A total of 46 patients (76.1% males, 35/46) were included in this analysis and contributed 133 meropenem Css. Patient’s demographic and clinical characteristics are reported in Table 1. Median (IQR) age, weight, and serum creatinine were 58.5 (54.0–67.0) years, 70.0 (60.0–80.0) kg, and 0.7 (0.4–1.2) mg/dL, respectively. Overall, hospital-acquired pneumonia and intra-abdominal infections accounted for the majority of indications for meropenem treatment (60.8%, 28/46 patients). Overall, median GFR was significantly different when using mCL_CR_ compared to eGFR_CKDEPI_, eGFR_CG_, and eGFR_MDRD_ (74.7 mL/min vs. 103.1 mL/min/1.73 m^2^ vs. 112.6 mL/min/1.73 m^2^ vs. 108.5 mL/min/1.73 m^2^, *p* < 0.001). No difference was observed in the median eGFR values obtained by means of the three empiric formulas. AKI was observed in 28.3% (13/46) of the subjects, and 26.1% of patients (12/46) had at least an episode of ARC.

**Figure 1:**
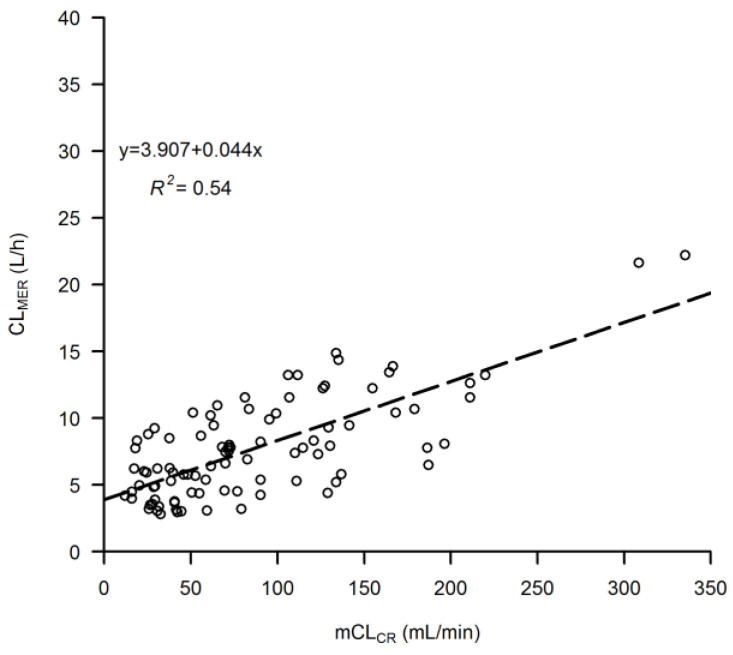
Linear regression between meropenem clearance (CL_M_) vs. measured creatinine clearance (mCL_CR_). The dashed line represents the line of regression.

**Figure 2:**
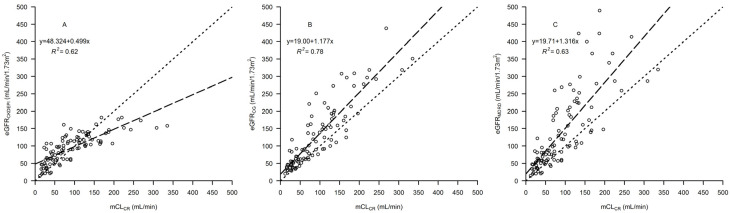
Linear regressions between (**A**) estimated glomerular filtration rate (eGFR) calculated by means of the CKDEPI formula (eGFR_CKDEPI_) vs. measured creatinine clearance (mCL_CR_), (**B**) eGFR estimated by means of the Cockcroft–Gault formula (eGFR_CG_) vs. mCL_CR_ and (**C**) eGFR estimated by means of the MDRD formula (eGFR_MDRD_) vs. mCL_CR_. The dashed lines represent the line of regression. The dotted lines are the identity lines.

**Figure 3:**
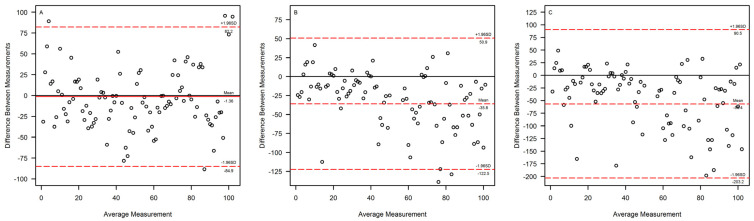
Bland–Altman plot for assessing the agreement between (**A**) measured creatinine clearance (mCL_CR_) vs. estimated glomerular filtration rate (eGFR) calculated by means of the CKDEPI formula (eGFR_CKDEPI_), (**B**) mCL_CR_ vs. eGFR estimated by means of the Cockcroft–Gault formula (eGFR_CG_), and (**C**) mCL_CR_ vs. eGFR estimated by means of the MDRD formula (eGFR_MDRD_). The red dashed lines represent the average difference and the 95% C.I. for the average difference.

Linear regression between CL_M_ vs. mCL_CR_ is shown in Figure 1. Linear regressions between eGFR_CKDEPI_ vs. mCL_CR_, eGFR_CG_ vs. mCL_CR_, and eGFR_MDRD_ vs. mCL_CR_ are shown in Figure 2. Bland-Altman plots for assessing the agreement between mCL_CR_ vs. eGFR_CKDEPI_, mCL_CR_ vs. eGFR_CG_, and mCL_CR_ vs. eGFR_MDRD_ are presented in Figure 3. eGFR_CG_ showed a better correlation with mCL_CR_ (R^2^ = 0.78), compared to those of eGFR_CKDEPI_ vs. mCL_CR_ and eGFR_MDRD_ vs. mCL_CR_ (R^2^ = 0.62 and 0.63, respectively). Both eGFR_CG_ and eGFR_MDRD_ overestimated mCL_CR_ across all ranges of renal function, while eGFR_CKDEPI_ overestimated mCL_CR_ up to 90 mL/min, then underestimated it.

**Figure 4:**
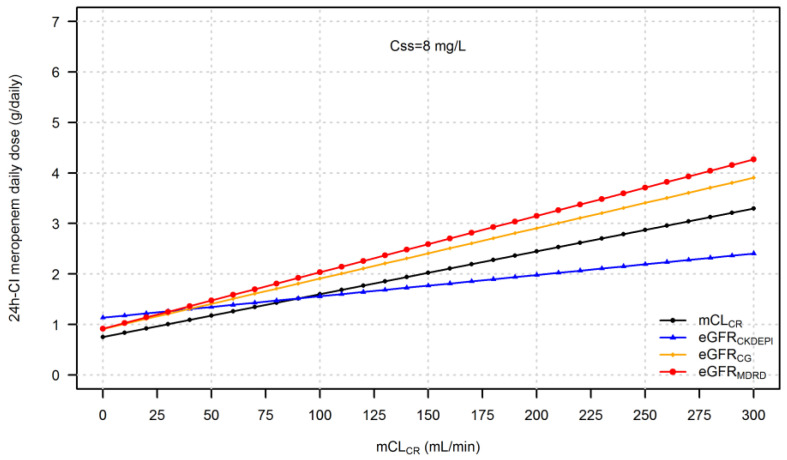
24 h-CI meropenem daily dose necessary to achieve the targeted C_ss_ of 8 mg/L by using eGFR_CKDEPI_, eGFR_CG_, or eGFR_MDRD_ compared to mCL_CR_.

**Figure 5:**
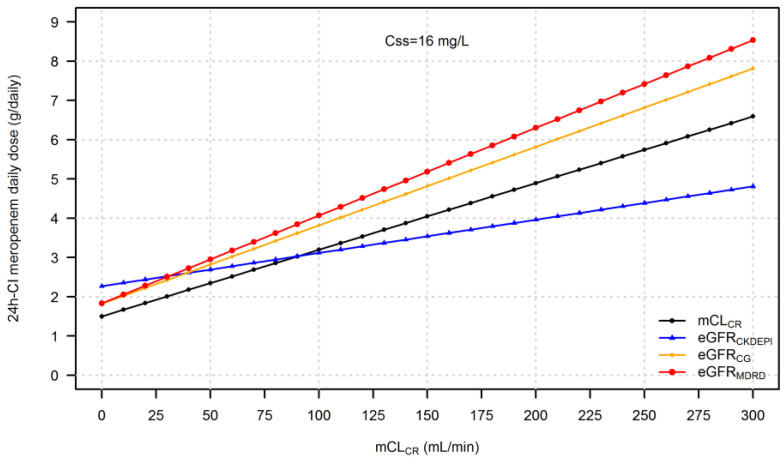
Twenty-four h-CI meropenem daily doses are necessary to achieve the targeted C_ss_ of 16 mg/L by using eGFR_CKDEPI_, eGFR_CG_, or eGFR_MDRD_ compared to mCL_CR_.

The daily dose of 24 h-CI meropenem needed to attain a PK/PD target of Css/MIC of 4–8 considering the EUCAST clinical breakpoint of meropenem against Enterobacterales and *P. aeruginosa* (namely, Css of 8 or 16 mg/L) based on IR-eGFR_CKDEPI_, IR-eGFR_CG_, and IR-eGFR_MDRD_ are depicted in Figure 4 and Figure 5, respectively.

Meropenem daily dosages based on eGFR equations were consistently different from those based on mCL_CR_. When GFR was calculated by means of eGFR_CG_ or eGFR_MDRD_, higher than necessary doses were estimated due to an overestimation of mCL_CR_. Similarly, this occurs when using eGFR_CKDEPI_ in patients with mCL_CR_ < 90 mL/min. Table 2 reports the median difference in meropenem daily dose (in g/daily) when using eGFR_CKDEPI_, eGFR_CG_, and eGFR_MDRD_ with respect to mCL_CR_.

## 4. Discussion

This is the first study that assessed the performances of commonly empirical formulas for eGFR estimation in determining meropenem dosages that are optimal for the empirical treatment of Gram-negative infections in critically ill patients.

For hydrophilic antibiotics that are eliminated mainly unmodified by the renal route, such as meropenem, a high correlation between creatinine clearance and drug clearance was described in different patient populations [13,19]. The existence of such a relationship is of utmost importance for clinicians, as it allows them to adjust drug dosage based on the degree of a patient’s renal function [13]. In our patients, measured creatinine clearance was linearly associated with CL_M_, but it could account for no more than 54% of the variability of meropenem elimination. This is plausible, considering that meropenem is also eliminated by tubular secretion [20] and that normal physiology is greatly modified in critically ill patients so that the pharmacokinetics of antibiotics predominantly cleared by the renal route may be highly variable. Consistent with our observation is that reported by a recent prospective study conducted among 25 critically ill patients with sepsis who were treated with three h-extended infusion meropenem every 8 h [21]. The correlation between CL_M_ and mCL_CR_ was even lower than ours, the R^2^ ranging 0.23–0.30 according to the time of the pharmacokinetic assessment after starting therapy.

Different studies assessed the performances of eGFR equations compared to mCL_CR_ across different ranges of GFR, and almost all showed important flaws when using such mathematical equations for renal function estimation in critically ill patients [22,23,24,25]. A recent retrospective study conducted on 237 critically ill patients in Arabia with a mean mCL_CR_ of 102.7 ± 65.4 mL/min showed that eGFR_CKDEPI_, eGFR_CG_, and eGFR_MDRD_ had an accuracy as low as 12.7–30% in estimating mCL_CR_ within ±10%, and that both eGFR_CG_ and eGFR_MDRD_, but not eGFR_CKDEPI_, were significantly biased. Moreover, that study confirmed an overestimation of all equations in patients with AKI and in patients with ARC, an overestimation of eGFR_CG_, and an underestimation of eGFR_CKDEPI_ [26]. GFR-estimating equations showed poor performances both in patients with AKI and ARC. In the former scenario, eGFR formulas performed poorly when compared to mCL_CR_, with a bias ranging from 7.4 to 11.6 mL/min [27]. On the contrary, in the context of ARC, eGFR equations have been shown to generally underestimate mCL_CR_ [28]. We can confirm this finding for eGFR_CKDEPI_, but we observed an overestimation, especially for the eGFR_MDRD_ in our cohort. In this regard, it should be noted that the MDRD equation was validated only for patients with impaired or modestly impaired renal function (eGFR < 60 mL/min/1.73 m^2^), and its use should not be extended to patients of higher classes of renal function.

Collectively, these data clearly indicate that in critically ill patients, renal function should be measured rather than estimated, especially for those experiencing ARC [28]. For drugs that are eliminated mainly by the kidneys, the implications of a proper assessment of renal function are of utmost importance for drug dosing. From our findings, it emerges that in critically ill patients, estimation of meropenem dosages should be based on mCL_CR_. The use of empirical formulas should be discouraged, as it may lead to an underestimation of the daily maintenance dose with the consequent high risk of meropenem underexposure if eGFR_CKDEPI_ is used, or to an overestimation of the drug dose if eGFR_CG_ or eGFR_MDRD_ are used. However, it is worth noting that nowadays the optimal administration of beta-lactams in critically ill patients should be supported by TDM, and results should be interpreted by clinical pharmacologists with experience in antimicrobial and infectious diseases. In a recent experience of antimicrobial TDM in critically ill patients, we reported the need for a dose increase based on TDM for meropenem in 13.5% of cases and a dose decrease for piperacillin-tazobactam in 44% of patients [29].

In critically ill patients the attainment of an aggressive pharmacodynamic target of efficacy for beta-lactams has been shown effective both for achieving a positive clinical outcome from the infectious episode and for preventing the development of resistance. Specifically, a recent retrospective study conducted among 74 critically ill patients who received 24 h-CI meropenem for the treatment of different infections between December 2020 and July 2021 showed that achieving a Css/MIC ≥ 4.63 was associated with a clinical cure [10]. Another retrospective study conducted among 116 critically ill patients who received CI meropenem, piperacillin, or ceftazidime for the treatment of documented Gram-negative infections showed that targeting a C_ss_/MIC ratio > 5 for these beta-lactams could prevent microbiological failure and/or resistance development [11].

We are aware of the presence of some limitations in this study. First, our data were retrospectively collected, and this only allowed us to get sparse pharmacological and laboratory data. Second, the sample size was quite limited due to the need for both meropenem plasma concentrations and mCL_CR_. Third, we applied the empirical formulas to all ranges of renal function, which may be inaccurate in some circumstances. A strength of our analysis was that the continuous infusion mode of administration gave us the opportunity to exactly calculate CL_M_ in each patient and to associate this pharmacokinetic variable to different estimates of renal function.

## 5. Conclusions

In conclusion, we showed all the eGFR equations are not adequate for calculating the doses of 24 h-CI meropenem that are needed to attain optimal pharmacodynamic targets of efficacy in critically ill patients. Clinicians should rely on mCL_CR_ and TDM for optimizing the 24 h-CI meropenem dose in empiric therapy against susceptible Gram-negative pathogens in the critically ill population.

## Figures and Tables

**Table 1:** Demographic and clinical characteristics of the population (n = 46).

Variable		Median or Count	IQR Range or %
Age (yrs)		58.5	54–67
Gender (male/female)		35/11	76.1/24.9
Body weight (kg)		70.0	60.0–80.0
BMI (kg/m^2^)		24.2	21.7–26.8
Assessement of renal function			
	Serum creatinine	0.7	0.4–1.2
	mCL_CR_ (mL/min)	74.7	40.5–129.3
	eGFR_CKDEPI_ (mL/min/1.73 m^2^)	103.1	62.6–126.7
	eGFR_CG_ (mL/min/1.73 m^2^)	112.6	61.7–185.2
	eGFR_MDRD_ (mL/min/1.73 m^2^)	108.5	58.9–207.0
	Patients with AKI	13	28.3
	Patients with ARC	12	26.1
Reason for meropenem			
	IAI	18	39.1
	HAP	10	21.7
	Sepsis/septic shock	9	19.6
	BSI	6	13.1
	Others	3	6.5
Meropenem treatment			
	Dose (g q24h by CI)	2.0	2.0–4.0
	Treatment duration (days)	12.0	8.0–19.0
	Css (mg/L)	13.4	9.4–19.5
	Clearance (L/h)	7.8	5.3–11.6

ARC, augmented renal clearance (defined as mCL_CR_ ≥ 130 mL/min); AKI, acute kidney injury (defined as mCL_CR_ < 30 mL/min); BMI, body mass index; BSI, bloodstream infection; Css, meropenem steady-state concentration; eGFR_CG_ estimated glomerular filtration rate calculated by means of the Cockcroft–Gault formula; eGFR_CKDEPI_ estimated glomerular filtration rate calculated by means of the CKDEPI formula; eGFR_MDRD_ estimated glomerular filtration rate calculated by means of the MDRD formula; HAP, hospital acquired pneumonia; IAI, intra-abdominal infections; mCL_CR_, measured creatinine clearance. Data are presented as median (IQR) for continuous variables and as a number (%) for categorical variables.

**Table 2:** Differences in meropenem dose amount (in g/daily) when using eGFR formulas compared to mCL_CR_, for targeting Css at 8 and 16 mg/L.

mCL_CR_	Css = 8 mg/L			Css = 16 mg/L		
	eGFR_CKDEPI_	eGFR_CG_	eGFR_MDRD_	eGFR_CKDEPI_	eGFR_CG_	eGFR_MDRD_
10	0.34	0.18	0.19	0.68	0.36	0.38
30	0.26	0.21	0.25	0.52	0.42	0.50
60	0.13	0.25	0.33	0.26	0.50	0.66
90	0.00	0.30	0.41	0.00	0.60	0.82
120	−0.13	0.34	0.49	−0.26	0.68	0.97
150	−0.26	0.39	0.57	−0.52	0.78	1.14
180	−0.38	0.43	0.65	−0.76	0.86	1.30
210	−0.51	0.48	0.73	−1.02	0.96	1.46
240	−0.64	0.52	0.81	−1.28	1.04	1.62
270	−0.76	0.57	0.89	−1.52	1.14	1.78
300	−0.89	0.61	0.97	−1.78	1.22	1.94

## Data Availability

Not applicable.
